# Mendel's First Law: partisan interests and the parliament of genes

**DOI:** 10.1038/s41437-022-00545-x

**Published:** 2022-06-11

**Authors:** Carl Veller

**Affiliations:** 1 Center for Population Biology, University of California, Davis, CA USA; 2 Department of Evolution and Ecology, University of California, Davis, CA USA

**Keywords:** Evolutionary genetics, Population genetics

## Abstract

Mendel’s First Law requires explanation because of the possibility of ‘meiotic drivers’, genes that distort fair segregation for selfish gain. The suppression of drive, and the restoration of fair segregation, is often attributed to genes at loci unlinked to the drive locus—such genes cannot benefit from drive but do suffer its associated fitness costs. However, selection can also favour suppressors at loci linked to the drive locus, raising the question of whether suppression of drive usually comes from linked or unlinked loci. Here, I study linked and unlinked suppression in a two-locus model with initial stable polymorphism at the drive locus. I find that the invasion rate of suppressors is a decreasing function of the recombination fraction between the drive and suppressor loci. Surprisingly, the relative likelihood of unlinked vs. linked suppression increases with the strength of drive and is insensitive to the fitness costs of the driver allele. I find that the chromosomal position of the driver influences how rapidly it is suppressed, with a driver in the middle of a chromosome suppressed more rapidly than a driver near the tip. When drive is strong, only a small number of chromosomes are required for suppression usually to derive from unlinked loci. In contrast, when drive is weak, and especially when suppressor alleles are associated with fitness costs, suppression will usually come from linked loci unless the genome comprises many chromosomes.

## Introduction

Mendel’s First Law, that of fair segregation, holds across most genomic loci in most of the sexual species in which segregation patterns have been characterized. This wide jurisdiction is in spite of genes, called meiotic drivers, that distort segregation in their own favour, gaining a selfish transmission advantage that allows them to spread through a population even when they reduce organismal fitness (Prout 1953; Lindholm et al. 2016; Burt and Trivers 2006).

Compounding this threat to Mendel’s First Law, theory has shown that genes at other loci can be under selection to distort segregation at a linked polymorphic locus (Liberman 1976; Thomson and Feldman 1976). It is therefore facially surprising that Mendel’s First Law holds so widely. Indeed, Bell (1982) even entertained the possibility that ‘alleles which distort segregation ratios will almost always increase at the expense of those which do not; that as a consequence most genomes are saturated with such alleles; and that the Mendelian ratios usually observed do not result from a virtual absence of segregation distortion, but from the fact that all chromosomes are very nearly balanced for alleles which, relative to the aboriginal wild-type, cause extreme distortion’ (pg. 439).

With the aim of rescuing Mendel’s First Law from these theoretical attacks, Eshel (1985) showed that, while linked modifiers can be selected to distort segregation at a polymorphic locus, unlinked modifiers are always under selection to usher segregation at the locus back towards Mendelian proportions [similar results had been obtained by Leigh (1971) and Thomson and Feldman (1976)]. The reason is that genes at unlinked loci cannot gain a transmission advantage from drive but do suffer its associated fitness costs. In species with many chromosomes, unlinked loci will generally outnumber linked loci for any given focal locus, constituting a ‘parliament of genes’ (Leigh 1971), ‘the overwhelming majority of which will combine against any member which pursues its selfish advantage at the expense of the common interest of the whole’ (Leigh 1987, pg. 244). This ‘parliamentary’ explanation of the stability of Mendel’s First Law has found considerable influence, with Crow (1991), for example, believing it to be ‘mainly responsible for keeping the Mendelian system honest.’

However, modifiers at linked loci also can be selected to restore Mendelian segregation at a focal locus (Liberman 1976), and there is reason to believe that their ‘partisan interests’ are stronger than the ‘parliamentary interests’ of unlinked suppressors—after all, linked suppressors not only suffer the fitness costs associated with the driving allele, but can also themselves be driven against. Therefore, it is unclear whether suppression of drive is usually furnished by the majority of unlinked loci at which selection for suppression is weak, or the minority of linked loci at which selection for suppression is strong.

This question has gained fresh importance with the proposal and development of synthetic gene drives for the control of pest and pathogen populations (Burt, 2003; Esvelt et al. 2014). A key concern is that gene drives will be thwarted by suppressor genes that arise in the populations they are engineered to manage (Unckless et al. 2017; Gomulkiewicz et al. 2021). It is therefore vital to fully understand the nature of selection in favour of suppressors across the genome, and how it is affected by the selective parameters induced by various gene drive designs.

Here, I study the prospects of linked and unlinked suppression in a simple, two-locus model with initial polymorphism at the drive locus owing to recessive fitness costs of the drive allele. This model is relevant to a number of well-known examples of meiotic drive (Burt and Trivers 2006). Its deterministic dynamics have been studied extensively [e.g., Prout et al. (1973), Hartl (1975), Thomson and Feldman (1976), Feldman and Otto (1991)], leading to a comprehensive classification of the conditions under which distorters and restorers of Mendelian segregation can invade, and the equilibria that obtain when they do. However, the question of the relative likelihood of linked vs. unlinked suppression is a probabilistic one, which analysis of deterministic dynamics is not suited to answer. Instead, I take a stochastic approach, characterizing the relative invasion rates of suppressors at various recombination distances from the drive locus.

## Methods

### Model

The model species is a cosexual diploid, with random mating in a population of size *N*. At the ‘drive locus’, there segregate two alleles, *D* and *d*. The drive allele *D*, when unsuppressed, is transmitted to a fraction (1 + *δ*)/2 of the gametes of *Dd* heterozygotes, where *δ* > 0 is the ‘strength of drive’.

In the absence of suppression, stable polymorphism at the drive locus requires that *D* be costly and that *DD* homozygotes suffer disproportionately large fitness costs (Prout 1953). To limit the number of parameters, I assume that *Dd* and *dd* individuals have equal fitness, relative to which *DD* homozygtes have reduced fitness 1 − *s*. In this case, if *δ* < *s* (assumed throughout), the system evolves to a stable polymorphism at which *D*’s frequency is *p* = *δ*/*s* and mean fitness is 1 − *p*^2^*s* (Prout 1953).

With the drive locus initially held at this stable polymorphism, a single suppressor allele *M* appears by mutation at a locus elsewhere in the genome. The recombination fraction between the drive and suppressor loci is *r* ≤ 1/2. *M* has no direct effect on fitness, but restores Mendelian segregation in *Dd* heterozygotes, and in this effect is dominant with respect to the ancestral allele at its locus, *m*.

The quantity of primary interest is the probability *E* that *M* escapes stochastic loss (‘establishes’), as a function of *r*, *δ*, *s*, and the genotype in which *M* first appears (*dM*/*dm*, *DM*/*dm*, *Dm*/*dM*, or *DM*/*Dm*). This quantity will mainly be contrasted across different recombination fractions *r*, so I will write it as *E*(*r*). I assume that suppressors appear by mutation sufficiently infrequently that the fate of *M*—establishment or loss—will almost certainly be resolved before the appearance of another suppressor. This assumption allows focus to be limited to the two-locus system.

### Simulations

Wright–Fisher simulations of the above model were run for various configurations of *δ*, *s*, *r*, and *M*’s initial genotype, with a population size of *N* = 10,000. For each configuration, as many trials were run as could be completed within a set computational time window (24 h CPU time); the number of trials therefore varied across configurations, but was always large enough that standard errors were negligibly small.

Each trial proceeded until (i) *M* was lost, (ii) *D* was lost, or (iii) *M* attained a frequency greater than 99% (since *M* is dominant, complete fixation could take considerably longer). I scored events (ii) and (iii) as ‘establishment’ of *M*, ignoring the possibility of *D* being lost by drift (i.e., independent of suppression). To ensure that *d* and *D* were seldom lost by drift, I focused on parameter regimes where 0.2 < *p* < 0.8.

For each configuration of *δ*, *s*, and *r*, an average establishment probability of *M*, *E*, was calculated by weighting the estimates obtained for the four initial genotypes *dM*/*dm*, *DM*/*dm*, *Dm*/*dM*, and *DM*/*Dm* by (1−*p*)^2^, *p*(1 − *p*), *p*(1 − *p*), and *p*^2^ respectively.

### Analytical calculations

To obtain analytical estimates of the average establishment probability of *M* in certain parameter cases, I calculate the proportionate rate of increase of *M* when rare, ${s}_{M}=(\varepsilon ^{\prime} -\varepsilon )/\varepsilon$, where *ε* ≪ 1 is the frequency of *M* in one generation and $\varepsilon ^{\prime}$ its expected frequency in the next. I then approximate the establishment probability of *M* as *E* ≈ 2*s*_*M*_ (Haldane 1927). Note that, in cases where *s*_*M*_ must be calculated separately for different initial *M* genotypes, the overall establishment probability is simply a linear average across the distinct *s*_*M*_ values.

## Results

### Suppression at the drive locus

First, consider the case where the suppressor allele *M* appears by mutation at the drive locus itself (*r* = 0), or at a locus very tightly linked to the drive locus (*r* ≈ 0). If *M* initially appears alongside the drive allele *D* (with probability *p*), it forms a permanent (or nearly so), costly, non-driving haplotype that is almost certain to go extinct.

If, instead, *M* appears alongside *d* (with probability 1 − *p*), it forms a haplotype that is never driven against, and which always (or at least for very many generations) resides in individuals with fitness 1 relative to the population average 1 − *p*^2^*s*. In this case, *M* therefore has a selective advantage

1 $${s}_{M}=\frac{{p}^{2}s}{1-{p}^{2}s}\approx {p}^{2}s$$

and establishes with probability ~ 2*p*^2^*s*.

The average establishment probability of *M* when *r* ≈ 0 is therefore

2 $$E(0)\approx p\times 0+(1-p)\times 2{p}^{2}s=2{p}^{2}(1-p)s.$$

**Fig. 1:**
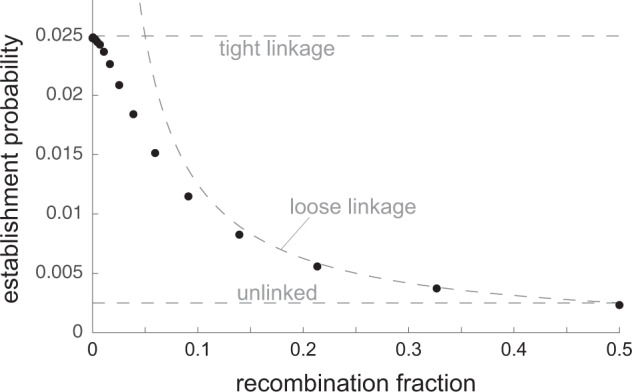
Average establishment probability of a suppressor allele appearing at various recombination distances from the drive locus. Dots are values from simulations; dashed lines are analytical predictions [Eqs. (2), (5), and (7)]. Parameters: *s* = 0.1, *δ* = 0.05.

This approximation agrees well with estimates obtained from simulations (Figs. 1, S1).

Suppose that the mutation rate to suppressor alleles at the drive locus (or at a given tightly linked locus) is *μ*_*l*_ per gamete, so that the population supply at the locus is 2*Nμ*_*l*_ per generation. Then the rate at which suppressors establish (their ‘invasion rate’) at the locus is approximately

3 $$I(0)=2N{\mu }_{l}\times E(0)\approx 4N{\mu }_{l}{p}^{2}(1-p)s.$$

### Suppression at an unlinked locus

If *M* appears at a locus unlinked to the drive locus (*r* = 1/2), it enjoys a selective advantage because, by reducing its co-transmission with the costly *D* allele from *Dd* heterozygotes, it is transmitted to fitter offspring on average (Eshel 1985; Crow 1991). Quantitatively, this advantage depends on the proportion of *Dd* heterozygotes, 2*p*(1 − *p*), *M*’s reduction in co-transmission with *D* from *Dd* heterozygotes, *δ*/2, and the average fitness cost to *D*-inheriting offspring, *ps*. *M*’s suppression of drive in the parental generation therefore increases the average fitness of offspring to whom it is transmitted by an amount ~*p*^2^(1 − *p*)*δs*.

In more precise terms, *M* spreads because its suppression of drive generates negative linkage disequilibrium (LD) between itself and the costly *D* allele (Appendix 1; Gomulkiewicz et al.^ 2021^). The fitness boost to *M*-bearing offspring calculated in the paragraph above reflects the amount of LD created by suppression in the parental generation alone. Across generations, new LD is created by suppression and old LD is destroyed by recombination. When selection and drive are weak, the per-generation increment in LD due to suppression is approximately constant, and, with free recombination between the drive and suppressor loci, LD between *M* and *D* rapidly approaches an asymptotic value of about twice the per-generation increment [Appendix 1; similar results have been obtained in different contexts by Brandvain and Coop (2012) and Muralidhar et al. (2021)]. Therefore, in this case, the selective advantage to *M* is approximately twice the increase in offspring fitness caused by a single generation of suppression:

4 $${s}_{M}\approx 2{p}^{2}(1-p)\delta s.$$

*M*’s establishment probability at a locus unlinked to the drive locus (*r* = 1/2) is therefore approximately

5 $$E(1/2)\approx 2{s}_{M}\approx 4{p}^{2}(1-p)\delta s,$$

which agrees well with simulations (Figs. 1, S1), particularly when drive is strong relative to selection (Fig. S1). If the mutational supply of suppressors at a given unlinked locus is 2*Nμ*_*u*_ per generation, the invasion rate of suppressor alleles at the locus is

6 $$I(1/2)\approx 2N{\mu }_{u}\times 4{p}^{2}(1-p)\delta s=8N{\mu }_{u}{p}^{2}(1-p)\delta s.$$

Comparing Eqs. (2) and (5), we find that the ratio of a suppressor allele’s establishment probabilities at an unlinked vs. a tightly linked locus is ~ 2*δ*. It is surprising that this ratio is independent of *s* and increasing in *δ*, since, by common interpretation, the ‘interests’ of unlinked suppressors are in restoring population mean fitness while the primary advantage to linked suppressors is the avoidance of drive. By this interpretation, we might expect the ratio of invasion rates of unlinked vs. linked suppressors to increase with *s* and decrease with *δ* (Price et al. 2020).

From the same comparison, we see that the establishment probability of a suppressor at a tightly linked locus is greater than at an unlinked locus if *δ* < 1/2. That is, the ‘partisan’ interests of a linked suppressor are effectively stronger than the ‘parliamentary’ interests of an unlinked suppressor only if drive is sufficiently weak. This threshold value of *δ* is especially relevant since, when drive is limited to the meiosis of one sex (as it usually is), the value of *δ* for the species, averaged across the two sexes, is necessarily ≤1/2.

Taking into account the relative numbers of linked and unlinked loci, Eqs. (3) and (6) reveal the conditions under which unlinked suppression is more likely than linked suppression in the special case of a species with no crossing over. Suppose that the haploid genome of the species comprises *n* chromosomes of equal physical length. Then the overall invasion rate of linked suppressors is proportional to 4*Nμ*_*l*_*p*^2^(1 − *p*)*s*, while the overall invasion rate of unlinked suppressors is proportional to 8*Nμ*_*u*_(*n* − 1)*p*^2^(1 − *p*)*δs*. Assuming the per-locus rate of appearance of suppressor alleles to be the same at linked and unlinked loci (*μ*_*l*_ = *μ*_*u*_), unlinked suppression is seen to be more likely than linked suppression if 2(*n* − 1)*δ* > 1, i.e., if *n* > 1 + 1/(2*δ*). This calculation can be generalized to allow for unequal chromosome lengths (Appendix 3).

### Intermediate recombination rates

Figure 1 displays simulation estimates of the average establishment probabilities of suppressors at various recombination distances from the drive locus, for the case *s* = 0.1, *δ* = 0.05. Results for other parameter configurations are displayed in Fig. S1. The average establishment probability is seen to be a decreasing function of the recombination fraction between the suppressor and drive loci. This implies that, all else equal, (i) suppression of drive will be more rapid in species with fewer chromosomes, and (ii) the number of chromosomes in the haploid set must be greater than two for suppression usually to be unlinked. Note that linked modifiers of the segregation ratio can also be under selection to distort, rather than restore, Mendelian segregation at a polymorphic locus [e.g., Liberman (1976), Thomson and Feldman (1976)], so (i) and (ii) should not be interpreted as predictions of how common drive is in species with smaller or larger karyotypes.

To understand the forces governing establishment probabilities at various recombination distances, recall that, because *M* is a Mendelian allele with no direct effect on fitness, it can spread only via LD with *d* (Gomulkiewicz et al. 2021). With this in mind, two recombination ‘regimes’ can be distinguished, depending on the primary source of LD between *M* and *d*. In the first regime, the suppressor locus is tightly linked to the drive locus. Following a standard heuristic argument [e.g., Desai and Fisher (2007)], an *M* allele coupled with *d* is almost certain to establish if it attains a copy number of 1/(2*s*_*M*_) which, since *M*’s dynamics below this copy number are dominated by drift, takes ~1/(2*s*_*M*_) generations on average. Therefore, if a copy of *M* is unlikely to recombine away from its associated *d* allele in 1/(2*s*_*M*_) generations (*r* ≪ 2*s*_*M*_), it behaves as if perfectly linked to *d* in its crucial establishment phase. The establishment probability of suppressors at these recombination distances is therefore similar to that of a perfectly linked suppressor given in Eq. (2): *E*(*r*) ≈ 2*p*^2^(1 − *p*)*s* (Fig. S1). In this regime, the LD that is relevant to *M*’s establishment is the LD created by its chance initial appearance alongside *d* or *D*.

The second regime is where the suppressor and drive loci are sufficiently loosely linked that, in *M*’s establishment phase, its initial LD with *d* or *D* is rapidly destroyed by recombination, so that the LD relevant for *M*’s establishment is instead the LD generated by *M*’s suppression of drive. In this regime, when selection and drive are weak, LD between *M* and *d* rapidly approaches a ‘quasi-equilibrium’ value (derived in Appendix 1) which confers on *M* a selective advantage *s*_*M*_ ≈ *p*^2^(1 − *p*)*sδ*/*r* and therefore an establishment probability

7 $$E(r)\approx 2{p}^{2}(1-p)s\delta /r.$$

Eq. (7) can be accurate for a fair range of recombination fractions (Figs. 1,S1).

### The chromosomal position of the driver

In the simulations, the establishment probability of *M* was estimated for a necessarily sparse sample of recombination fractions *r* between the suppressor and drive loci. To estimate the establishment probability *E*(*r*) as a smooth function of *r*, I interpolated these simulation estimates using a cubic spline. This enables calculation, for arbitrary genetic maps and genomic positions of the drive locus, of the overall invasion rate of suppression and its likely genomic source.

I first address the importance of the chromosomal position of the drive locus. Consider a genome with a single chromosome and a uniform genetic map of length *L* Morgans. Suppose further that there is no crossover interference, so that genetic distances *l* translate to recombination fractions via Haldane’s map function, $r=R(l)=[1-\exp (-2l)]/2$ (Haldane 1919). If the drive locus is situated at position *x* ϵ [0, 1] along the normalized length of the chromosome, then the average establishment probability of a suppressor appearing by mutation somewhere along the chromosome—assuming each position to be equally likely—is

8 $$\int\nolimits_{0}^{1}E\left(R(| x-y| )\right)dy.$$

**Fig. 2:**
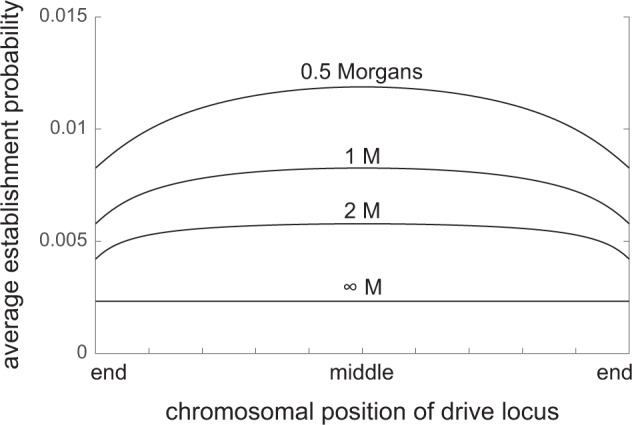
For various positions of the drive locus along a chromosome, the average establishment probability of a linked suppressor appearing at a random position on the chromosome (with each position assumed to be equally likely). Results are displayed for several genetic lengths of the chromosome, assuming a uniform recombination rate and no crossover interference. Parameters: *s* = 0.1, *δ* = 0.05.

Figure 2 displays numerical calculations of Eq. (8) when *s* = 0.1 and *δ* = 0.05. The position of the drive locus is seen to affect the average establishment probability of linked suppressors: distorters in the middle of the chromosome are more readily suppressed than distorters at the end. The reason is that a central distorter has tightly linked loci on either side of it, and a maximum genetic distance to another locus on the chromosome of half the chromosome’s length; in contrast, a distal distorter has tightly linked loci to only one side, and the maximum distance to another locus is the chromosome’s full length. Therefore, the average recombination fraction with a randomly placed suppressor locus is smaller for central than for distal drive loci, and so the average establishment probability of suppressors is higher in the former case. Since recombination relations to unlinked loci do not depend on a locus’s chromosomal position, this argument extends to genomes with more than one chromosome: suppression of centrally located distorters should be more rapid than suppression of distally located distorters.

### The party or the parliament?

**Fig. 3:**
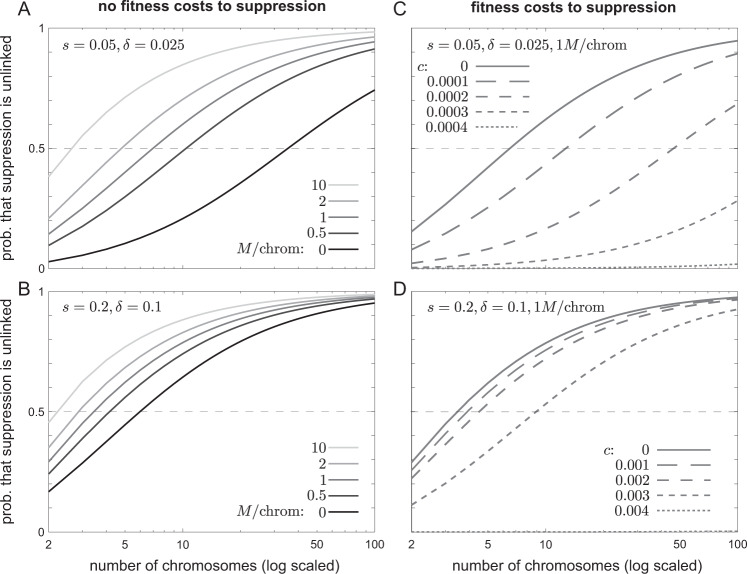
The likelihood that suppression of drive derives from loci unlinked to the drive locus, rather than from linked loci, for various chromosome numbers and per-chromosome genetic lengths. Chromosomes are assumed to be equally sized—relaxing this assumption will tend to increase the number of chromosomes required for suppression usually to be unlinked, for reasons explained in the text.

The interpolated establishment probability function *E*(*r*) also enables calculation, for various linkage maps, of the relative likelihood of linked vs. unlinked suppression. Figure 3 displays the likelihood that suppression is unlinked rather than linked given various numbers of (equally sized) chromosomes in the genome, assuming that the per-locus mutation rate to suppressor alleles is constant across the genome. When drive is strong (Fig. 3B), only a few chromosomes are required for unlinked suppression to be more likely than linked suppression—i.e., for the parliament usually to enforce Mendel’s First Law. In contrast, when drive is weak (Fig. 3A), many chromosomes can be required for suppression usually to be unlinked—in such cases, for some species, Mendel’s First Law will tend to be policed by the party, not the parliament, of genes. Consistent with the comparison of unlinked and tightly linked suppressors above, the relative likelihood of unlinked suppression increases with the strength of drive, *δ*, and is insensitive to the fitness cost of the driver, *s* (Fig. S2).

These results ignore several factors which might affect the relative likelihood of linked vs. unlinked suppression. First, I have assumed, unrealistically, that all chromosomes in the genome are of equal size. For combinatorial reasons, heterogeneity of chromosome sizes will increase the fraction of locus pairs that are linked (Veller et al. 2019), and thus increase the importance of linked suppression relative to unlinked suppression.

Second, I have assumed that the suppressor allele *M* completely restores Mendelian segregation in *Dd* heterozygotes. However, in natural drive systems, suppression is often incomplete [e.g., Didion et al. (2015)]. Suppose that *M* reduces the fraction of gametes to which *D* segregates in *Dd* heterozygotes from (1 + *δ*)/2 to $(1+\delta ^{\prime} )/2$, with $\delta ^{\prime} \,<\, \delta$. In Appendix 1, I show that incomplete suppression ($\delta ^{\prime} \,>\, 0$) reduces the establishment probabilities of both tightly and loosely linked suppressors by an approximately equivalent fraction, $1-\delta ^{\prime} /\delta$, relative to complete suppression. This suggests that incomplete suppression will not substantially affect the relative likelihood of linked vs. unlinked suppression.

Third, I have assumed that *M*’s only effect is on the segregation ratio at the drive locus. In particular, I have assumed that *M* does not directly affect the fitness of its bearers. However, fitness costs to suppressor alleles are known in a number of drive systems [e.g., Wu et al. (1989)], and will clearly influence the likelihood that suppressor alleles establish. [Fitness costs to suppression can also lead to complicated dynamics once a suppressor allele has established (Charlesworth and Hartl 1978; Haig and Grafen 1991)].

Suppose that a rare suppressor *M*, were it not costly, would show a proportionate rate of increase *s*_*M*_, but that it instead exerts a fixed, additive fitness cost *c*_*M*_ on its bearers. Then its proportionate rate of increase, taking its costs into account, is ~ *s*_*M*_ − *c*_*M*_, so that its establishment probability in a large population is approximately 2(*s*_*M*_ − *c*_*M*_), provided *s*_*M*_ > *c*_*M*_. Since *s*_*M*_ is greater for linked than for unlinked suppressors, there are three possibilities (assuming that the costs of suppression are independent of its genomic source): (i) *c*_*M*_ is so large that linked suppressors cannot invade (*c*_*M*_ > *p*^2^*s*); therefore, neither can unlinked suppressors invade; (ii) *c*_*M*_ is large enough to preclude invasion of unlinked suppressors, but not tightly linked suppressors (roughly, 2*p*^2^(1 − *p*)*δs* < *c*_*M*_ < *p*^2^*s*); (iii) *c*_*M*_ is not large enough to prevent unlinked suppressors from invading (*c*_*M*_ < 2*p*^2^(1 − *p*)*δs*). In case (i), suppression never evolves. In case (ii), suppression necessarily derives from loci linked to the drive locus. In case (iii), the fitness cost *c*_*M*_ reduces the establishment probabilities of linked and unlinked suppressor alleles by about the same amount −2*c*_*M*_, but this represents a proportionately greater reduction for unlinked suppressors (which are more weakly selected). Therefore, *c*_*M*_ increases the relative likelihood of linked vs. unlinked suppression.

Overall, then, fitness costs to suppressor alleles increase the relative likelihood of linked vs. unlinked suppression, and therefore increase the number of chromosomes required for suppression usually to derive from unlinked loci. This effect can be quite drastic even for modest fitness costs to suppression (Fig. 3C, D).

Finally, it is worth noting that, for heterogametic species in which the sex-specific chromosome is degenerate or absent, if segregation distortion occurs in the heterogametic sex in favour of the non-degenerate sex chromosome—as it commonly does (Burt and Trivers 2006)—then suppression must necessarily derive from loci unlinked to the drive locus, since suppressor alleles on the non-degenerate sex chromosome can only prevent their own preferential segregation.

## Discussion

The maintenance of Mendel’s First Law is often attributed to the action of genes unlinked to the loci at which segregation distortion arises. This is because of the obvious selective advantage unlinked suppressors enjoy and the fact that the majority of the genome is usually unlinked to any given drive locus (Eshel 1985; Crow 1991). However, selection can also favour suppression at loci linked to the drive locus, and this selection is typically stronger than for unlinked suppressors (Fig. 1). Here, I have studied the relative invasion rates of linked and unlinked suppressors, in a model where initial polymorphism at the drive locus is ensured by fitness costs to driver homozygotes.

Surprisingly, I find that the relative likelihood of unlinked suppression increases with the strength of drive, *δ*, and is insensitive to the strength of selection against drive homozygotes, *s*. This conflicts with the conventional interpretation of how selection acts on linked and unlinked suppressors, on the basis of which one might expect the relative likelihood of unlinked suppression instead to decrease with *δ* and to increase with *s* (Price et al. 2020). The underlying reason for this result is that the strength of selection in favour of an unlinked suppressor depends on the degree of negative LD the suppressor generates with the costly drive allele and the fitness costs it avoids because of this negative association. LD builds up because, by suppressing drive, the suppressor allele reduces its co-transmission with the drive allele; the degree of LD thus generated is proportional to the strength of drive, *δ*. The fitness costs avoided are proportional to the strength of the fitness costs of the driver, *s*, and so the advantage to the unlinked suppressor is proportional to *δs*, with *δ* appearing in this expression because of its effect on LD, not because the unlinked suppressor avoids being driven against.

In contrast, a suppressor in tight linkage with the non-drive allele does avoid being driven against, but this does not affect its selective advantage—the suppressor is simply a Mendelian allele in permanent linkage with the high-fitness allele at the drive locus (Gomulkiewicz et al. 2021). Its selective advantage is therefore proportional to *s*, so that the ratio of the strengths of selection in favour of unlinked vs. linked suppression is proportional to *δs*/*s* = *δ*.

I have also found that the chromosomal position of the driver affects how rapidly it is suppressed, with centrally located drivers suppressed more rapidly than distal drivers (Fig. 2). The reason is that selection in favour of more tightly linked suppressors is stronger, and centrally located loci have tighter average linkage relations to other loci on their chromosome than distally located loci do. Centromeres, the chromosomal sites where the segregation machinery attaches during mitotic and meiotic cell divisions, have long been suspected to be subject to meiotic drive (Henikoff et al. 2001). The result above predicts that suppression of centromere drive will be more rapid in metacentric species (with centromeres towards the middle of the chromosomes) than in acrocentric species (with centromeres at or near the ends of the chromosomes), all else equal.

Finally, I have found, in most parameter configurations of the model, that when suppression of drive is costless, only a few chromosomes are required in the haploid set for suppression usually to be furnished by unlinked loci (Fig. 3A, B), supporting the common view that the ‘parliament of genes’ is responsible for the maintenance of Mendel’s First Law (Leigh 1971; Crow 1991). In contrast, when suppression is costly, many chromosomes can be required for suppression usually to be unlinked to the drive locus (Fig. 3C, D).

### The source of polymorphism at the drive locus

For the segregation ratio at a locus to be of interest, the locus must obviously be polymorphic. In the model I have studied, there is initially stable polymorphism at the drive locus owing to recessive fitness costs of the driver which prevent its fixation. This fitness scheme obtains in several prominent drive systems, such as *Segregation Distorter* in *Drosophila melanogaster* (Temin and Marthas 1984), the *t*-haplotype in mice (Lyon 1986), the Ab10 neocentromere in maize (Higgins et al. 2018), and the centromeric *D* haplotype in monkeyflowers (Fishman and Saunders 2008; Fishman and Kelly 2015).

An alternative source of stable polymorphism that has received theoretical attention in the segregation distortion literature is overdominance (Liberman 1976; Eshel 1985). The empirical relevance of this fitness scheme for meiotic drive is unknown, although the low rates of recombination around centromeres might induce associative overdominance in these regions (Becher et al. 2020; Gilbert et al. 2020). The calculations developed in this paper can readily be adapted to the case of overdominance at the drive locus (Appendix 2). In the symmetric overdominance case, where *Dd* heterozygotes have fitness 1 + *s* relative to *dd* and *DD* homozygotes, the drive allele *D* is initially held in a stable polymorphism at frequency *p* = (*s* + *δ* + *sδ*)/2*s*, assuming *δ* < *s*/(1 + *s*). The average establishment probability of a suppressor that subsequently appears by mutation at the drive locus (*r* = 0) is

9 $$E(0)\approx \frac{(s+\delta )(s-\delta )\delta }{2{s}^{2}},$$

while the average establishment probability of an unlinked suppressor (*r* = 1/2) is

10 $$E(1/2)\approx \frac{(s+\delta )(s-\delta ){\delta }^{2}}{{s}^{2}}.$$

The ratio of establishment probabilities of unlinked and tightly linked suppressors in this case is therefore *E*(1/2)/*E*(0) = 2*δ*, as in the case of recessive fitness costs to the driver [Eqs. (2) and (5)]. This suggests that the results obtained for recessive fitness costs in this paper could be robust to other fitness schemes that maintain initial polymorphism at the drive locus.

Under many other fitness schemes, however, the driver is expected to spread to fixation if unsuppressed (Prout 1953). Polymorphism at the drive locus is transient in this case, so that a suppressor allele has only limited time to establish and attain high enough frequency to impede the driver’s spread. I have obtained expressions for the average establishment probability of suppressors in this case, and will present them elsewhere [see also Unckless and Clark (2015), Gomulkiewicz et al. (2021)].

### Mutational biases and the mechanism of suppression

The comparisons in this paper assume that the rate of mutation to suppressor alleles is uniform across the genome—in particular, suppressors are equally likely to appear at a locus on the same chromosome as the drive locus and at a locus on a different chromosome. This assumption focuses attention on the selective forces promoting suppression in different regions of the genome; i.e., the relative strengths of the ‘partisan’ interests of linked suppressors and the ‘parliamentary’ interests of unlinked suppressors.

However, the assumption is unlikely to hold in many empirical instances of meiotic drive. For example, a theme that has emerged from recent molecular characterization of suppressors in several drive systems is that suppression often occurs via RNA interference [e.g., Dawe et al. (2018), Svedberg et al. (2021); reviewed in Price et al. (2020)]. This mechanism favours homologous (or ‘allelic’) suppression, and therefore biases the mutational supply of suppressors towards linked loci. Similarly, when drivers spread via the targeting of homologous sequence (as in many synthetic gene drive systems), suppression is disproportionately likely to occur via mutation to the target sequence itself (Gomulkiewicz et al. 2021).

On the other hand, when suppression is constrained to certain sites in the genome but drive can occur on any chromosome, suppressors appearing at a given site will usually be unlinked to the driver they suppress. The centromere drive hypothesis (Henikoff et al. 2001) posits that the rapid evolution of kinetochore proteins is due to suppression of meiotic drive at centromeres. If a new driver emerges at the centromere of a given chromosome, suppression can evolve at a linked locus only if the relevant kinetochore protein happens to be encoded on the same chromosome; more likely, suppression will be unlinked in this case.

Finally, in assuming that the mutational appearance of suppressor alleles follows the establishment of stable polymorphism at the drive locus, I have implicitly assumed that the mechanism of suppression is tailored to the driver in question. However, the regular occurrence of meiotic drive at various locations in the genome can ultimately select for alterations of the genetic system that inhibit drive in general, such as to the recombination rate (Haig and Grafen 1991; Haig 2010; Brandvain and Coop 2012), the degree of inbreeding (Bull 2017; Martinossi-Allibert et al. 2021), and the mechanics of meiosis itself (Haig 2010).

### Supplementary information

Supplementary Figures S1 and S2

## Appendix 1

Here, I show that the expected rate of increase of the suppressor allele *M* depends on linkage disequilibrium (LD) between *M* and the driver allele *D*. I then use this relationship to show that, under deterministic dynamics when selection and drive are weak relative to recombination (*δ* < *s* ≪ *r*) and *M* is rare, the value of LD between *D* and *M* approaches 1/*r* times the per-generation increment in LD generated by suppression; in particular, when *r* = 1/2, LD approaches twice the per-generation increment.

Let the frequency of *M* in a given generation be *ε*, assumed to be small. The frequency of *D* is initially *p* = *δ*/*s*, and stays very close to this value since *M* is rare. The recombination fraction between *M* and *D* is *r*. Let *f*_*D**M*_, *f*_*d**M*_, *f*_*D**m*_, and *f*_*d**m*_ denote the frequencies of the four possible haplotypes in this generation. Denote by *C* (for ‘covariance’) the degree of LD between *M* and *D*:

$$C={f}_{DM}-p\varepsilon ={f}_{dm}-(1-p)(1-\varepsilon )=-{f}_{dM}+(1-p)\varepsilon =-{f}_{Dm}+p(1-\varepsilon )={f}_{DM}{f}_{dm}-{f}_{dM}{f}_{Dm}.$$

The frequencies of the *M*-containing haplotypes in gametes (and therefore in the next generation, since mating is random) are given by

$$\begin{array}{rcl}\bar{w}{f}_{DM}^{\prime}&=&{f}_{DM}^{2}(1-s)+2{f}_{DM}{f}_{dM}\frac{1}{2}+2{f}_{DM}{f}_{Dm}(1-s)\frac{1}{2}+\,2{f}_{DM}{f}_{dm}(1-r)\frac{1}{2}+2{f}_{dM}{f}_{Dm}r\frac{1}{2}\\ &=&{f}_{DM}({f}_{DM}+{f}_{dM}+{f}_{Dm}+{f}_{dm})-{f}_{DM}({f}_{DM}+{f}_{Dm})s+({f}_{dM}{f}_{Dm}-{f}_{DM}{f}_{dm})r\\ &=&{f}_{DM}-{f}_{DM}ps-Cr\\ &=&p\varepsilon (1-ps)+C(1-ps-r);\\ \bar{w}{f}_{dM}^{\prime}&=&{f}_{dM}^{2}+2{f}_{dM}{f}_{DM}\frac{1}{2}+2{f}_{dM}{f}_{Dm}(1-r)\frac{1}{2}+2{f}_{dM}{f}_{dm}\frac{1}{2}+2{f}_{DM}{f}_{dm}r\frac{1}{2}\\ &=&{f}_{dM}({f}_{dM}+{f}_{DM}+{f}_{Dm}+{f}_{dm})+\,({f}_{DM}{f}_{dm}-{f}_{dM}{f}_{Dm})r\\ &=&{f}_{dM}+Cr\\ &=&(1-p)\varepsilon -C(1-r),\end{array}$$

where $\bar{w}=1-{p}^{2}s$. The change in frequency of *M* is therefore

$${\varepsilon }^{\prime}={f}_{DM}^{\prime}+{f}_{dM}^{\prime}=\frac{1}{1-{p}^{2}s}\left[p\varepsilon (1-ps)+C(1-ps-r)+\,(1-p)\varepsilon -C(1-r)\right]=\varepsilon -C\frac{ps}{1-{p}^{2}s},$$

so that *M*’s rate of spread is

11 $${s}_{M}=\frac{{\varepsilon }^{\prime}-\varepsilon }{\varepsilon }=-\frac{C}{\varepsilon }\cdot \frac{ps}{1-{p}^{2}s}.$$

The change in LD between *M* and *D* is

12 $$\begin{array}{rcl}{C}^{\prime}={f}_{DM}^{\prime}-p{\varepsilon }^{\prime}&=&{\displaystyle\frac{p\varepsilon (1-ps)+C(1-ps-r)}{1-{p}^{2}s}-p\left(\varepsilon -C\frac{ps}{1-{p}^{2}s}\right)}\\ &=&{\displaystyle\frac{-{p}^{2}(1-p)\varepsilon s+C[1-r-p(1-p)s]}{1-{p}^{2}s}}\\ &\approx &-{p}^{2}(1-p)\varepsilon s+C(1-r),\end{array}$$

so that $C^{\prime} -C\approx -{p}^{2}(1-p)\varepsilon s-rC$, where the first term is the increment to LD from *M*’s suppression of drive, and the second term is the destruction of old LD by recombination.

Since *δ*, *s* ≪ 1, *ε* changes slowly over time, so that − *p*^2^(1 − *p*)*ε**s* can be assumed to be constant. In that case, setting LD to zero in the first generation (*C*_0_ = 0), Eq. (12) defines the sequence

$$\begin{array}{rcl}{C}_{1}&=&-{p}^{2}(1-p)\varepsilon s,\\ {C}_{2}&=&-{p}^{2}(1-p)\varepsilon s+{C}_{1}(1-r)={C}_{1}+{C}_{1}(1-r),\\ {C}_{3}&=&-{p}^{2}(1-p)\varepsilon s+{C}_{2}(1-r)={C}_{1}+{C}_{1}(1-r)+{C}_{1}{(1-r)}^{2},\ldots \end{array}$$

which converges at rate *r* to the value *C*_*∞*_ = *C*_1_/*r* = − *p*^2^(1 − *p*)*ε**s*/*r*. At this equilibrium value of LD, the strength of selection in favour of *M* is, from Eq. (11),

13 $${s}_{M}=-\frac{{C}_{\infty }}{\varepsilon }\cdot \frac{ps}{1-{p}^{2}s}\approx \frac{{p}^{3}(1-p){s}^{2}}{r}=\frac{{p}^{2}(1-p)s\delta }{r},$$

where the last step follows because *p* = *δ*/*s*. When *r* = 1/2 in particular, LD rapidly increases towards a value of *C*_*∞*_ = − 2*p*^2^(1 − *p*)*ε**s*, at which

14 $${s}_{M}\approx 2{p}^{2}(1-p)\delta s,$$

which is Eq. (4) in the main text.

The calculations above can easily be extended to the case where *M* only partially suppresses drive. Suppose that *D* segregates to a fraction $(1+\delta ^{\prime} )/2$ of the gametes of *D**d* heterozygotes who carry a rare suppressor allele *M*, with $0\le \delta ^{\prime} < \delta$. Then $\bar{w}=1-{p}^{2}s$, and

$$\begin{array}{rcl}\bar{w}{f}_{DM}^{\prime}&=&p\varepsilon [1-ps+(1-p){\delta }^{\prime}]+C[1-ps+(1-p){\delta }^{\prime}-r(1+{\delta }^{\prime})],\\ \bar{w}{f}_{dM}^{\prime}&=&(1-p)\varepsilon (1-p{\delta }^{\prime})-C[1-p{\delta }^{\prime}-r(1-{\delta }^{\prime})],\end{array}$$

so that

$${\varepsilon }^{\prime}=\varepsilon +C\frac{(1-2r){\delta }^{\prime}-ps}{1-{p}^{2}s},$$

and

$$\begin{array}{rcl}{C}^{\prime}&=&{\displaystyle\frac{\varepsilon p(1-p)({\delta }^{\prime}-ps)+C[1-r+p(1-p)s+{\delta }^{\prime}(1-2p)(1-r)]}{1-{p}^{2}s}}\\ &\approx &\varepsilon p(1-p)({\delta }^{\prime}-ps)+C(1-r).\end{array}$$

So $C^{\prime} -C\approx \varepsilon p(1-p)(\delta ^{\prime} -ps)-Cr$, and thus, since $\delta ^{\prime} -ps=\delta ^{\prime} -\delta < 0$, negative LD accumulates between *M* and *D*, approaching at rate ~ *r* an asymptotic value of ${C}_{\infty }\approx \varepsilon p(1-p)(\delta ^{\prime} -ps)/r$. Therefore,

$${s}_{M}=-\frac{{C}_{\infty }}{\varepsilon }\cdot \frac{ps}{1-{p}^{2}s}\approx \frac{p(1-p)(ps-\delta ^{\prime} )\delta }{r},$$

which is decreasing in ${\delta }^{\prime}$. If *M* appears at a locus unlinked to the drive locus (*r* = 1/2), then ${s}_{M}\approx 2p(1-p)(ps-{\delta }^{\prime})\delta$, so that *M*’s establishment probability

$$E(1/2)\approx 4p(1-p)(ps-{\delta }^{\prime})\delta .$$

If, instead, the partial suppressor *M* appears at a locus tightly linked to the drive locus, then (i) if *M* appears alongside *D* (with probability *p*), it forms a haplotype that does not drive with sufficient strength to compensate for its fitness costs, and therefore goes extinct; (ii) if *M* appears alongside *d*, it enjoys a selective advantage ${p}^{2}s-p\delta ^{\prime}$, and therefore establishes with probability ~ $2p(ps-\delta ^{\prime} )$, which is positive since $ps=\delta > \delta ^{\prime}$. Therefore, the average establishment probability of a suppressor allele appearing at a locus tightly linked to the drive locus (*r* ≈ 0) is

$$E(0)\approx p\times 0+(1-p)\times 2p(ps-{\delta }^{\prime})=2p(1-p)(ps-{\delta }^{\prime}).$$

Comparing the establishment probabilities of unlinked and linked suppressors, we find again that their ratio is ~ 2*δ*.

## Appendix 2

Suppose that the relative fitnesses of the *D**D*, *D**d*, and *d**d* genotypes are 1, 1 + *s*, and 1, and that *D* segregates to a fraction (1 + *δ*)/2 of the gametes of *D**d* individuals when it is unsuppressed. Then, if *δ* < *s*/(1 + *s*), there is a stable polymorphism at this locus in the absence of suppression, at which the frequency of *D* is *p* = (*s* + *δ* + *s**δ*)/2*s* (Liberman 1976). For reference, 1 − *p* = (*s* − *δ* − *s**δ*)/2*s* and 2*p* − 1 = (*δ* + *s**δ*)/*s*.

Consider first the case of a single suppressor allele *M* appearing by mutation at the drive locus. If it appears in concert with *D* (probability *p*), it will be eliminated. If, instead, it appears in concert with *d* (probability 1 − *p*), then it forms a Mendelian haplotype that resides in *D**d* individuals (fitness 1 + *s*) a fraction *p* of the time and *d**d* individuals (fitness 1) a fraction 1 − *p* of the time, so that its proportionate fitness advantage over the population average 1 + 2*p*(1 − *p*)*s* is

$${s}_{M}=\frac{[p(1+s)+(1-p)(1)]-[1+2p(1-p)s]}{1+2p(1-p)s}=\frac{p(2p-1)}{1+2p(1-p)s}s.$$

When *δ* and *s* are small,

$${s}_{M}\approx p(2p-1)s=\frac{(s+\delta +s\delta )(\delta +s\delta )}{2s}\approx \frac{(s+\delta )\delta }{2s},$$

so that the average establishment probability of *M* when *r* = 0, averaged across initial backgrounds, is

$$E(0)\approx (1-p)2{s}_{M}\approx \frac{(s+\delta )(s-\delta )\delta }{2{s}^{2}}.$$

Now consider the case where *M* appears by mutation at a locus unlinked to the drive locus. *M* gains an advantage because, from *D**d* heterozygotes [a fraction 2*p*(1 − *p*) of individuals], it reduces its co-transmission with *D* by *δ*/2. Offspring to whom *d* is transmitted have higher fitness than offspring to whom *D* is transmitted, by an amount [*p*(1 + *s*) + (1 − *p*)(1)] − [*p*(1) + (1 − *p*)(1 + *s*)] = (2*p* − 1)*s* on average. So the average fitness of the offspring of *M*-bearing individuals is increased by 2*p*(1 − *p*) × *δ*/2 × (2*p* − 1)*s* = *p*(1 − *p*)(2*p* − 1)*δ**s*. This single-generation advantage to *M* is due to a single-generation increment in positive LD between *M* and *d* caused by suppression of drive. As for the case of homozygous fitness costs of the driver (Appendix 1), when selection and drive are weak, the amount of LD between *M* and *d* increases towards an asymptotic value of twice the per-generation increment, so that the selective advantage to an unlinked suppressor is approximately twice the single-generation value calculated above:

$${s}_{M}\approx 2p(1-p)(2p-1)\delta s={\displaystyle\frac{(s+\delta +s\delta )(s-\delta -s\delta )(\delta +s\delta )\delta }{2{s}^{2}}\approx \frac{(s+\delta )(s-\delta ){\delta }^{2}}{2{s}^{2}}}.$$

The unlinked suppressor therefore establishes with probability

$$E(1/2)\approx 2{s}_{M}\approx \frac{(s+\delta )(s-\delta ){\delta }^{2}}{{s}^{2}}.$$

The ratio of average establishment probabilities of linked and unlinked suppression is therefore

$$\frac{E(1/2)}{E(0)}=2\delta ,$$

as for the case of homozygous fitness costs.

## Appendix 3

Suppose there are *n* chromosomes in the haploid set, and that chromosome *i*’s relative physical length is *l*_*i*_, such that ∑_*i*_*l*_*i*_ = 1. There is no crossing over. We randomly choose the drive and suppressor loci, with all possible locus pairs equally likely. The probability that the drive and suppressor loci lie on the same chromosome is then ${\sum }_{i}{l}_{i}^{2}$, and the probability that they lie on different chromosomes is $1-{\sum }_{i}{l}_{i}^{2}$. If they lie on the same chromosome, the recombination fraction between them is *r* = 0, and the establishment probability of the suppressor allele is *E*(0) ≈ 2*p*^2^(1 − *p*)*s* [Eq. (2)]. If they lie on different chromosomes, the recombination fraction between them is *r* = 1/2, and the establishment probability of the suppressor allele is *E*(1/2) ≈ 4*p*^2^(1 − *p*)*δ**s* [Eq. (5)]. The ratio of invasion rates of unlinked vs. linked suppression is therefore

15 $$\frac{\left(1-{\sum }_{i}{l}_{i}^{2}\right)E(1/2)}{\left({\sum }_{i}{l}_{i}^{2}\right)E(0)}=2\delta \frac{1-{\sum }_{i}{l}_{i}^{2}}{{\sum }_{i}{l}_{i}^{2}}.$$

With no crossing over, all genetic shuffling is due to random assortment of chromosomes at meiosis, and the average recombination fraction among locus pairs in the genome is $\bar{r}=\frac{1}{2}\left(1-{\sum }_{i}{l}_{i}^{2}\right)$(Veller et al. 2019). Eq. (15) then becomes

16 $$2\delta \frac{2\bar{r}}{1-2\bar{r}}.$$
