# Supplementary material for: Mendel's First Law: partisan interests and the parliament of genes
Source: Heredity (Edinb). 2022 Jun 11;129(1):48–55. doi: 10.1038/s41437-022-00545-x (PMC9273594; doi:10.1038/s41437-022-00545-x)
Supplement: Supplementary file 1 — Supplementary Figures S1 and S2 [file 41437_2022_545_MOESM1_ESM.pdf]

Supplementary material - Mendel's First Law: Partisan interests and the parliament of genes.

Figure S1

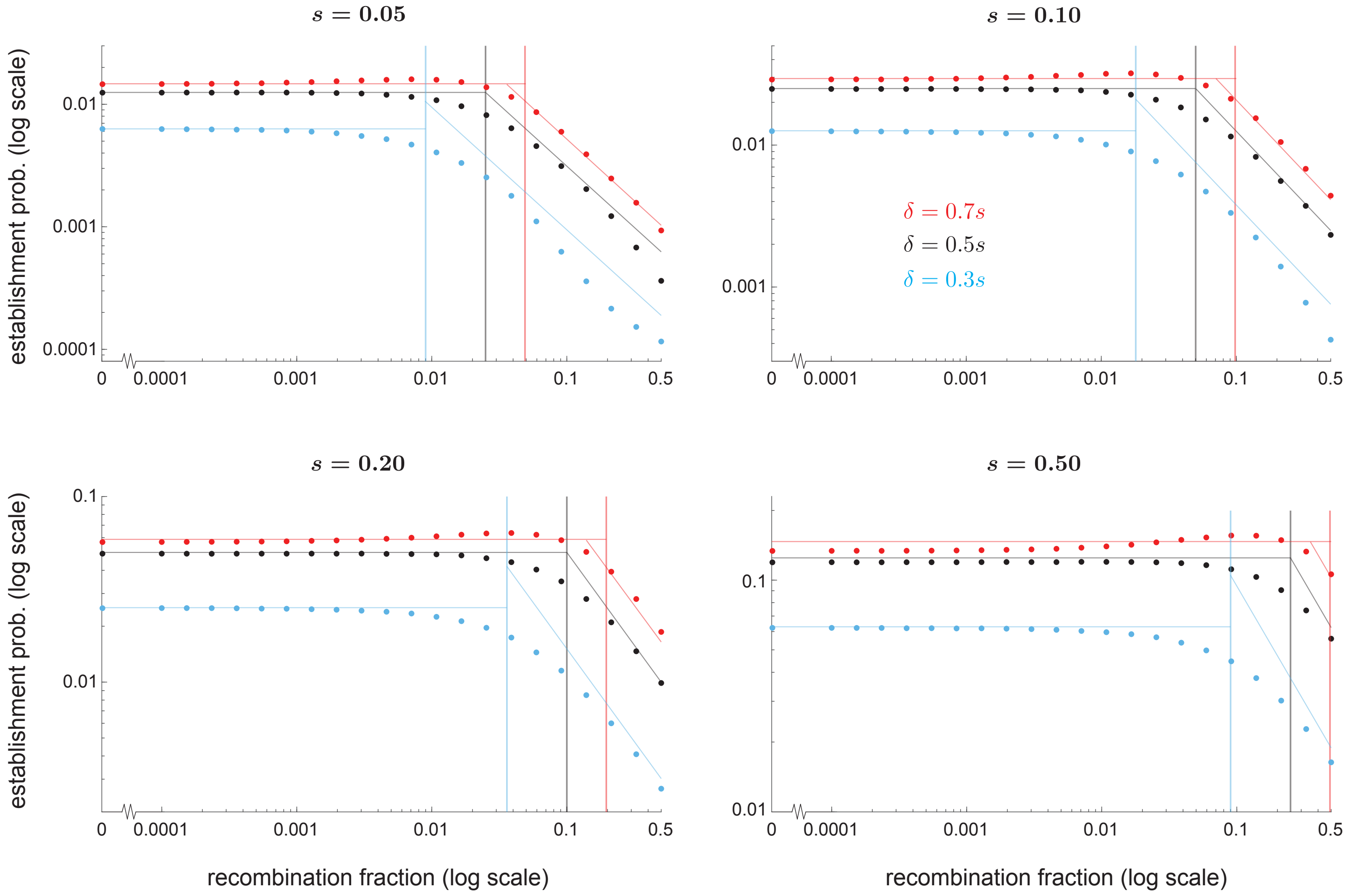

Average establishment probabilities of the suppressor allele M for various recombination fractions r between the suppressor and drive loci, and for various strengths of selection s and drive δ. Dots are values obtained from simulations. Vertical lines at $r = 2p^2s$ ($p = \delta/s$) are regime thresholds, to the far left of which we expect suppressors to behave as if perfectly linked to the drive locus, and to the far right of which we expect suppressors to behave as if loosely linked to the drive locus. Horizontal lines are analytical estimates of average establishment probabilities for tight linkage [Eq. (2)]; diagonal lines are analytical estimates of average establishment probabilities for loose linkage [Eq. (7)], with their bottom right tips (at $r = 1/2$) corresponding to analytical estimates for unlinked suppressors [Eq. (5)].

Figures S2

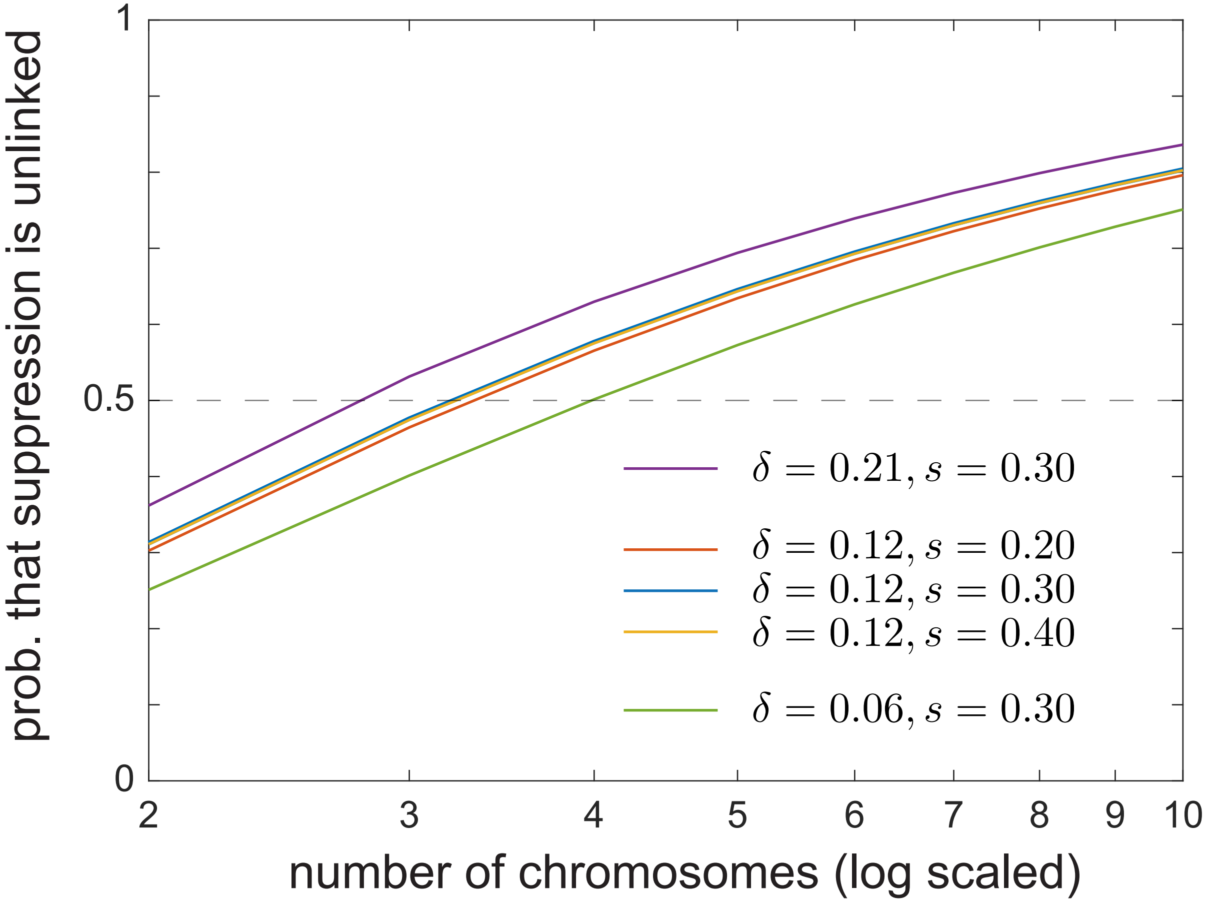

Relative likelihood that suppression of drive derives from loci unlinked to the drive locus for various chromosome numbers, strengths of drive δ, and strengths of selection s. The per-chromosome map length is 1 Morgan. The likelihood of unlinked suppression increases with δ when s is held constant (compare purple, blue, and green curves), but is insensitive to s when δ is held constant (compare red, blue, and yellow curves).
